# Dual-Temperature Microbiological Control of Cellular Products: A Potential Impact for Bacterial Screening of Platelet Concentrates?

**DOI:** 10.3390/microorganisms11092350

**Published:** 2023-09-20

**Authors:** Tanja Vollmer, Cornelius Knabbe, Jens Dreier

**Affiliations:** Herz- und Diabeteszentrum Nordrhein-Westfalen, Universitätsklinik der Ruhr-Universität Bochum, 32545 Bad Oeynhausen, Germany

**Keywords:** dual-temperature, platelet concentrates, bacterial safety

## Abstract

An experimental study by the Paul-Ehrlich Institute (PEI) demonstrated that temperatures between 35 and 37 °C are too high for the growth of some bacterial strains (e.g., *Pseudomonas fluorescens*), leading to false negative results. Thus, the question of whether it is necessary to adapt incubation temperatures for the microbiological control of blood products, especially platelet concentrates (PCs), to enhance safety and regulatory compliance has arisen. In order to further elucidate this issue, the growth capability of different bacterial strains of interest in PCs and the detection efficacy of cultivation of these at different incubation temperatures must be taken into account. Therefore, we inoculated PCs with 46 different strains (3–6 PCs from different donors per strain) from different origins (PC isolates, reference strains) and stored PCs at 20–22 °C under constant agitation. On day three of storage, the inoculated PCs were sampled; aerobic and anaerobic culture bottles (BacT/Alert AST/NST) were each inoculated with 5 mL of sample, and culture bottles were incubated at 25 and 35 °C using the automated BacT/Alert Dual-temperature system. Bacterial proliferation was enumerated using a colony-forming assay. All strains of *Enterobacteriacae* (*n* = 5), *Staphy-lococcus* spp. (*n* = 11), *Streptococcus* spp. (*n* = 5), and *Bacillus* spp. (*n* = 4) and most *Pseudomonas aeruginosa* strains (4 of 5) tested showed the capability to grow in most inoculated PCs, revealing a faster time to detection (TTD) at an incubation temperature of 35 °C. The tested *Pseudomonas putida* (*n* = 3) strains showed a noticeably reduced capability to grow in PCs. Nonetheless, those with a notable growth capability revealed a faster TTD at an incubation temperature of 35 °C. Only one of the four *Pseudomonas fluorescens* strains tested (strain ATCC 13525) was able to grow in PCs, showing a faster TTD at an incubation temperature of 25 °C but also detection at 35 °C. The commonly detected bacteria involved in the bacterial contamination of PCs showed a superior TTD at 35 °C incubation. Only one *P. fluorescens* strain showed superior growth at 25 °C; however, the microbiological control at 35 °C did not fail to identify this contamination. In conclusion, the use of PC screening using a dual-temperature setting for microbiological control is presently not justified according to the observed kinetics.

## 1. Introduction

The microbiological sterility testing of cell-based therapeutic preparations has been addressed regulatorily worldwide; guidelines in Europe were determined by the European Pharmacopoeia [1]. This directive has currently been revised and includes the parallel incubation of samples under aerobic (AE) conditions at incubation temperatures of 20–25 °C and a second sample under anaerobic (AN) conditions at incubation temperatures of 30–35 °C [1,2]. This chapter does not concern the examination of human blood or blood components, which is covered by Directive 2002/98/EC of the European Parliament and of the Council of 27 January 2003 and Commission Directive 2004/33/EC of 22 March 2004 implementing Directive 2002/98/EC [1]. However, the two incubation temperature frames required for the sterility testing of haematopoietic stem cells are in concordance with the observation that some bacterial species (e.g., *Pseudomonas fluorescens*) showed no proliferation at an incubation temperature above 34 °C or exclusively with high inocula and a significantly prolonged incubation time in an experimental study performed by the German National authorities at the Paul-Ehrlich Institute (PEI) [2]. As a result, the necessity of a possible adaption of incubation temperatures for the microbiological control of blood products is a subject of discussion. Mandatory monitoring of the bacterial contamination of blood components at the end of shelf life as part of routine quality control was defined in a national guideline (“Minimum Requirements for sterility control of blood components” Vote 43 [3]) in Germany and determined, inter alia, the sampling procedure (AE/AN cultivation, inoculation volume: 4–10 mL sample each, incubation at 30–37 °C for seven days [automated culture system]). Analogue guidelines were obligatory in other countries [4], and incubation temperatures of 35 °C were most often used.

Consequently, the microbiological control of platelet concentrates (PCs) may be falsely negative or accompanied with prolonged incubation times for some bacterial strains if incubation is only performed at 35–37 °C. This might be supported by the lack or underreporting of, for example, *Pseudomonas* species regarding haemovigilance data or bacterial screening studies ([5,6,7,8,9,10,11,12,13,14,15]). However, the question is whether it is a methodological lack of detection or simply the non-capability of species to grow in PCs at the required storage environment at room temperature under constant agitation.

To elucidate these two aspects, PCs from different donor origins were inoculated with a large number of bacterial isolates, including former PC isolates and strains from strain collections, frequently known as PC-contaminating species, as well as a large number of *Pseudomonas* species. The ability of the bacteria to proliferate in PCs, as well as the detection efficacy of microbiological cultivation in a dual-temperature approach, was monitored via the sampling of PCs at day three of storage. Bacterial proliferation was proven and enumerated via a colony-forming assay. The detection of the presence of bacterial contamination by microbiological cultivation was analysed by comparing the required time to detection (TTD) for the two incubation temperatures of 25 and 35 °C.

## 2. Material and Methods

### 2.1. PC Collection

Apheresis-derived single donor PCs were prepared after standard processing with the Haemonetics MCS+ (Haemonetics GmbH, München, Germany). Two PCs were prepared via double apheresis from a single donor (donor-related PCs, PC a/b). Donor arm disinfection was performed by spraying once with Kodan (Schuelke and Mayr, Norderstedt, Germany) and wiping with a sterile cotton swab. Extensive spraying was repeated once with a minimum liquid residence time of 90 s without further wiping. The PCs were stored in gas-permeable containers (LN994CF-CPP, Haemonetics GmbH, München, Germany) at 20–24 °C under constant agitation. The final product consisted of 2.0–4.0 × 10^11^ platelets/unit (205–295 mL) and 0.16–0.24 L/L ACD-A stabiliser and 0.76–0.84 L/L plasma per mL preparation.

### 2.2. Bacterial Strains

All of the strains used in this study (including their origins) are listed Table 1 and Table 2. Strains were isolates originally derived from contaminated PCs or reference stocks (ATCC strains [American Type Culture Collection, LGC Promochem GmbH, Wesel, Germany]) and Deutsche Sammlung von Mikroorganismen und Zellkulturen GmbH strains [Braunschweig, Germany]). The bacterial strain *Klebsiella pneumoniae* (PEI-B-08-08) was obtained from the PEI (Langen, Germany). The *Enterobacter aerogenes* strain L0120708 was provided by Verax Biomedical Inc. (Worcester, MA, USA).

### 2.3. Spiking Experiments, Microbiological Cultivation, and Bacterial Identification

PCs were spiked with a small titre (<100 colony-forming units (CFU)/bag) of each bacterial strain immediately after production to determine their growth capabilities (Table 1 and Table 2). The bacterial strains used for inoculation were grown overnight in trypticase soy broth (bioMérieux, Nürtingen, Germany) at 37 °C under aerobic conditions. For each strain, a master inoculation suspension was assessed via serial dilutions in Dulbecco’s phosphate-buffered saline (DPBS, Thermo Scientific, Waltham, MA, USA). For inoculation, 1 mL of an appropriate dilution was added to the PC samples. The bacterial inoculation titre was subsequently determined by plating 100 μL of an appropriate dilution of master suspensions on tryptone soya agar (Oxoid, Wesel, Germany) in triplicates for colony count. After incubation for 24 h, the number of colonies was counted, and the inoculation concentration of bacteria per ml of sample was calculated.

Potential influences on growth kinetics by donor-specific component variations were considered via the inoculation of three or up to five PCs from different donors. All of the PCs used were sampled before bacterial inoculation to assure baseline sterility. Sampling for the cultivation and enumeration of bacteria was performed during storage at 22 °C with constant agitation on day three after inoculation.

All artificially inoculated PCs were split to inoculate AE and AN culture bottles (BacT/Alert AST/NST, bioMérieux, Nürtingen, Germany), each with 5 mL of sample.

Culture bottles were incubated at 25 and 35 °C until a positive signal was detected or for up to seven days if they remained negative. Samples that did not react after seven days of storage were considered negative. Reactive culture bottles were subcultured on blood agar media (COS [bioMérieux, Nürtingen, Germany]), and isolates were identified using MALDI–ToF mass spectrometry (Bruker Daltonics, Bremen, Germany). A total of 100 µL aliquots of serial dilutions of PC samples were plated in triplicate onto tryptone soy agar and incubated at 37 °C for 48 h to monitor the growth kinetics of the bacterial strains. After incubation, the number of colonies was counted, and the bacterial count per mL of sample was calculated.

### 2.4. Statistical Analysis

All values are given as mean values (±standard deviation: SD). Mean values and SD were calculated, and correlation analysis was performed using GraphPad Prism 4.0 software (GraphPad Software, San Diego, CA, USA).

## 3. Results

### 3.1. Growth Characteristic of Gram-Negatives

**Figure 1:**
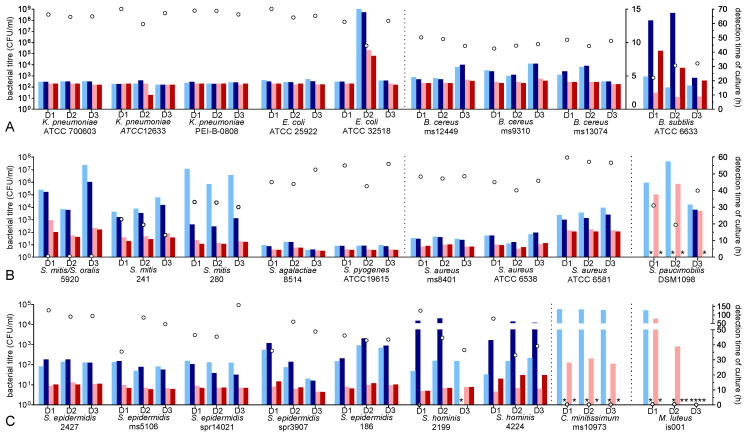
Bacterial proliferation of *Enterobacteriacea*, *Staphylococcus* spp., *Streptococcus* spp., and detection by culture at 25 °C and 35 °C incubation temperatures. For each bacterium, PCs from three different donors (D1–D3) were inoculated with bacteria listed in Table 1 and stored at 22 °C with agitation. (**A**) *Enterobacteriacea* and *Bacillus* spp., (**B**) *Streptococcus* spp., *Staphylococcus aureus* species. and *Sphingomonas paucimobilis*, (**C**) *Staphylococcus epidermidis* species, *Staphylococcus hominis* species, and two other bacterial species. Samples were taken on day three after inoculation. Bacteria were enumerated via colony-forming assay (white dots, left scale). The culture detection times are displayed in bars (right scale) separately for each culture bottle. The AE culture bottles at 25 °C are represented by the light blue bars, and the AN culture bottles at 25 °C are represented by the dark blue bars; the AE culture bottles at 35 °C are represented by the light red bars, and the AN culture bottles at 35 °C are represented by the dark red bars. The dotted vertical lines represent separators of species. * represents negative culture results for individual culture bottles.

All of the five Gram-negative strains, including the two species *K. pneumoniae* and *E. coli*, showed proliferation to high titres (*K. pneumoniae* mean 4.3 ± 3.0 × 10^8^ CFU/mL, range 4.3 × 10^7^–9.6 × 10^8^ CFU/mL; *E. coli* mean 4.3 ± 6.5 × 10^8^ CFU/mL, range 7.0 × 10^7^–1.5 × 10^9^ CFU/mL), independent from donors (Figure 1A, Table 1). The overall mean time to detection (TTD) was comparable for the AE and AN culture bottles at both incubation temperatures (*K. pneumoniae* 35 °C: mean 3.69 ± 0.39 h, 25 °C: 3.98 ± 0.20 h; *E. coli* 35 °C: mean 3.76 ± 0.07 h, 25 °C: 4.19 ± 0.15 h). The PC of donor 2 (inoculated with *E. coli* strain ATCC 35218) was excluded from our overall statistical analysis due to the lower mean growth of 4.9 × 10^5^ CFU/mL, leading to a prolonged incubation time for AE and AN cultivation and a delayed TTD at 25 °C (35 °C AE/AN: 8.83/7.98 h vs. 25 °C AE/AN: 15.02/14.52 h).

### 3.2. Growth Characteristic of Bacillus Species

The three *B. cereus* strains showed equivalent proliferation kinetics in different donors, with a mean titre of 1.6 ± 2.7 × 10^6^ CFU/mL (range 2.4 × 10^5^–8.5 × 10^6^ CFU/mL, Figure 1A, Table 1). The detection of bacteria is about 1.5 h faster using an incubation temperature of 35 °C (35 °C: mean 4.05 ± 0.21 h vs. 25 °C: 5.49 ± 0.93 h), and AE and AN culture bottles showed a comparable TTD. *B. subtilis* ATCC 6633 proliferated to a lower mean titre of 7.7 ± 6.5 × 10^3^ CFU/mL (range 7.0 × 10^2^–1.3 × 10^4^ CFU/mL, Figure 1A, Table 1). The TTD is about one and a half times faster using an incubation temperature of 35 °C (35 °C: mean 19.82 ± 13.00 h vs. 25 °C: 34.36 ± 23.78 h). However, the detection times of the AN culture bottles showed wider scattering.

### 3.3. Growth Characteristic of Staphylococcus Species

All of the *S. epidermidis* strains (*n* = 5) analysed were former isolates from contaminated PCs and also showed the capability to proliferate in PCs. The proliferation characteristics vary in a donor-dependent manner from 4.5 × 10^2^ to 9.4 × 10^4^ CFU/mL (mean 1.8 ± 2.6 × 10^4^ CFU/mL, Figure 1C, Table 1). The detection of bacterial contamination was more than twice as fast using an incubation temperature of 35 °C (mean 12.16 ± 1.67 h) compared to 25 °C (mean 28.85 ± 6.74 h). The detection times for the AE and AN culture bottles were comparable; detection was slightly faster in the AE bottle in some strains and vice versa. In total, *S. epidermidis* isolates showed more heterogenous kinetics among each other.

Comparable proliferation kinetics were observed for the two *S. hominis* PC isolates (titre mean: 1.2 ± 2.0 × 10^4^ CFU/mL, range 2.9 × 10^2^–4.9 × 10^4^ CFU/mL, Figure 1C, Table 1). The TTD was also considerably faster in the AE bottle when using an incubation temperature of 35 °C; the use of an AN culture bottle at an incubation temperature of 25 °C led to a significantly prolonged detection time or failure of detection.

The three different *S. aureus* strains showed proliferation to titres between 2.3 × 10^5^ and 9.5 × 10^7^ CFU/mL (mean 2.1 ± 3.2 × 10^7^ CFU/mL, Figure 1B, Table 1). A slightly faster TTD was observed in the AE culture bottle for both incubation temperatures; however, the TTD was generally nearly two times faster at an incubation temperature of 35 °C (mean 5.86 ± 1.58 h) compared to 25 °C (mean 9.82 ± 2.96 h). Interestingly, the TTD was longer for strain *S. aureus* ATCC 6581, although the corresponding titres were approximately two log phases higher.

### 3.4. Growth Characteristic of Streptococcus Species

The different *Streptococcus* isolates showed a very heterogeneous growth behaviour among themselves, although inter-donor differences were barely present by comparison (Figure 1B, Table 1). Isolate 5920 did not proliferate in any donor but was still detectable in culture with an approximately twice as fast TTD using a temperature of 35 °C. The other two *S. mitis* isolates were capable of proliferating (isolate 241: mean 5.2 ± 5.3 × 10^2^ CFU/mL, range 60–1.0 × 10^3^ CFU/mL; isolate 280: mean 2.0 × 10^4^ ± 8.6 × 10^3^ CFU/mL, range 1.0–1.6 × 10^4^ CFU/mL), and the TTD was two to four times faster at 35 °C compared to 25 °C (35 °C: 12.14 ± 3.73 h, 25 °C: 34.19 ± 11.36 h). *S. agalactiae* (mean 3.9 ± 5.3 × 10^6^ CFU/mL, range 7.3 × 10^5^–9.4 × 10^6^ CFU/mL) and *S. pyogenes* (mean 1.7 ± 1.5 × 10^7^ CFU/mL, range 4.8 × 10^5^–2.9 × 10^7^ CFU/mL) grew to higher titres, and the TTD was about 2 h faster for both isolates at 35 °C (4.63 ± 0.81 h vs. 6.75 ± 2.10 h at 25 °C), nearly independent of the culture condition used (AE or AN). Remarkably, bacterial contamination was detected earlier in the AN culture bottle for all *Streptococcus* spp.

### 3.5. Growth Characteristic of Pseudomonas Species

**Figure 2:**
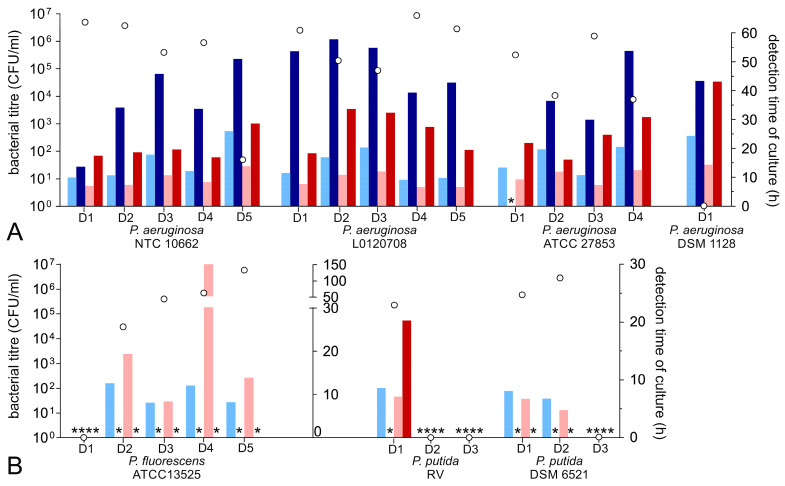
Bacterial proliferation of *Pseudomonas* spp. and detection by culture at 25 and 35 °C incubation temperature. For each bacterium, PCs from three to five different donors (D1–D5) were inoculated with the different *Pseudomonas* spp. listed in Table 2 and stored at 22 °C with agitation. (**A**) *P. aeruginosa*, (**B**) *P. fluorescens*, and *P. putida*. Samples were taken on day three after inoculation. The bacteria were enumerated via a colony-forming assay (white dots, left scale). The culture detection times are displayed in bars (right scale) separately for each culture bottle. The AE culture bottles at 25 °C are represented by the light blue bars, and the AN culture bottles at 25 °C are represented by the dark blue bars; the AE culture bottles at 35 °C are represented by the light red bars, and the AN culture bottles at 35 °C are represented by the dark red bars. * represents negative culture results for individual culture bottles.

The different *P. aeruginosa* isolates demonstrated diverse kinetics. Two strains (ATCC 9027 and DSM 1128) were not capable of proliferating in any donor. Only the DSM 1128 strain was still detectable in one donor after three days of incubation (donor 1, Figure 2A, Table 2). Regarding all other donors, those two strains were not detectable using incubation temperatures of 25 or 35 °C.

The remaining *P. aeruginosa* strains proliferated to a mean titre of 2.1 ± 2.7 × 10^6^ CFU/mL, range 7.9 × 10^3^–9.1 × 10^6^ CFU/mL. Bacterial contamination was generally detected earlier using the AE culture bottle; the AN bottle required about three times longer until a positive signal was reached, with a partly prolonged TTD of more than 40 h (Figure 2A, Table 2). Detection was considerably faster using an incubation temperature of 35 °C, and the TTD required in the AE bottle was 14.8 ± 5.0 h at 25 °C compared to 9.5 ± 2.5 h at 35 °C. The results regarding the PC samples from donor 5 inoculated with *P. aeruginosa* strain 10662 and from donor 1 inoculated with *P. aeruginosa* strain 1128 were excluded from these overall statistics, although the comparatively low titre did not result in considerable changes regarding the detection time.

The proliferation capacity of *P. putida* indicated donor dependency. If capable, strains grew to high titres of up to 1.2 ± 1.4 × 10^6^ CFU/mL in mean (range 2.2 × 10^5^–2.5 × 10^6^ CFU/mL). The detection of bacterial contamination was more efficient using the AE culture bottle. However, the TTD is faster at incubation temperatures of 35 °C (6.20 ± 1.25 h vs. 7.81 ± 0.93 h at 25 °C).

*P. fluorescens* strain ATCC 13525 was the only strain out of the four different *P. fluorescens* strains that showed the capability of growing in four out of five PCs from different donors (mean 1.8 ± 2.8 × 10^6^ CFU/mL, range 3.0 × 10^4^–5.9 × 10^6^ CFU/mL). The detection was only feasible using AE culture bottles. Remarkably, this was the only strain in this study to demonstrate a considerably prolonged incubation time at 35 °C. Bacterial contamination was already detected after 10.92 ± 3.62 h in mean at 25 °C, whereas a positive signal was first obtained after 8.27 h up to 148.90 h at 35 °C incubation temperature. However, the detection of bacterial contamination was also feasible using the higher incubation temperature.

### 3.6. Growth Characteristic of Other Species

*Sphingomonas paucimobilis* DSM 1098 was capable of growing in all inoculated PCs (Figure 1B, Table 2). Cultural detection was, with the exception of one sample, possible using the AE culture bottle. The mean TTD was lower at 35 °C incubation temperature (36.25 ± 8.15 h) compared to 25 °C (44.41 ± 13.00 h). *Corynebacterium minitissimum* isolate ms10973 was not capable of growing in different PCs but still detectable in all samples exclusively in the AE culture with a considerably lower mean TTD at 35 °C (28.64 ± 1.86 h vs. 122.8 ± 2.08 h at 25 °C). *M. luteus* was similarly not capable of growing in PCs, and if detectable, it was only detectable in AE culture bottles with a faster TTD at 35 °C.

The four *Cutibacterium* (formerly *Propionibacterium*) *acnes* strains—spr4694, spr4991, spr14472, and ATCC 11827—did not proliferate in the PCs. The presence of bacteria could not be detected at 25 °C incubation temperature after three days, and only two strains—spr4991 (115.65 h donor 1, 174.98 h donor 2) and ATCC 11827 (92.63 h donor 1, 88.95 h donor 2)—showed a positive culture result for the AN culture bottle at 35 °C.

## 4. Discussion

An experimental study by the PEI demonstrated that the standard incubation temperature of 35 °C (most often used for the screening of blood components for bacterial contamination) is too high for the growth of some bacterial strains that prefer or require lower temperatures for proliferation (e.g., *Pseudomonas fluorescens*, *Sphingomonas paucimobilis*) [17,18]. *Sphingomonas paucimobilis* showed a 9–24 h delayed detection time after the inoculation of 100–10,000 CFU into BacT/Alert culture bottles and no detection after the inoculation of 100 CFU at an incubation temperature of 36 °C compared to a reduced temperature of 32 °C. The *P. fluorescens* ATCC 13525 strain showed no proliferation at an incubation temperature above 34 °C (inoculation of 8 and 80 CFU per bottle) or only with high inocula and a significantly prolonged incubation time of ~200 h. Indeed, reducing the temperature to 32 °C resulted in reasonable detection times (8.5–32.7 h) after inoculation with a 8–8 × 10^6^ CFU/bottle. Independently from the inoculation titre, bacteria were not detected at 35 and 36 °C [2,17]. Manufacturers of automated culture systems studied this attempt and developed automated culture systems that work at two different temperatures (e.g., BacT/A) to fulfil the mandatory criteria of the European Pharmacopoeia for the microbiological sterility testing of cell-based therapeutic preparations [1]. The question is whether the extension of incubation temperatures to a dual-temperature approach could also extend the spectrum of bacteria that can be identified as contaminants of PCs. Does the usage of standard incubation at 35–37 °C result in false-negative results for some bacterial species, such as *Pseudomonas* spp.? Similarly, could the detection of other strains frequently known as contaminating organisms, such as *Enterobacteriacea*, *Staphylococcus* spp., *Streptococcus* spp., or *Bacillus* spp., be improved by using a second incubation temperature and lead to the benefit of an enhanced TTD? Regarding the second question, based on current data, the answer is clearly “no”. None of the isolates of the different species tested benefited from a reduced culture temperature. However, what about *Pseudomonas* spp. and *S. paucimobilis*?

Initially, one should take into account the data regarding the presence of *Pseudomonas* spp. as a contaminant of PCs and the relevance regarding transfusion-transmitted bacterial infections (TTBI). A summary of the results from the large BACON, SHOT, and BACTHEM studies disclosed the isolation of two *Pseudomonas* spp. isolates in red blood cells but did not mention the involvement of PCs [19]. Data from some large screening studies over the years have revealed no detection of *Pseudomonas* spp. [7,8,10]), whereas other studies have reported the isolation of *Pseudomonas* spp. as a rare event [9,11,12]. The German haemovigilance data from 1997 to 2020 reported only one non-fatal transfusion reaction with *Pseudomonas aeruginosa* (fresh frozen plasma), but no PCs were involved [5,13]. Two fatal TTBI with *P. aeruginosa* with PCs were reported to the US Food and Drug Administration from 1995 to 2004 [20], and two TTBI with PCs, either by *P. aeruginosa* (no severe case, absence of life-threatening or long-term threat) or *P. fluorescens* (severe, immediately life-threatening), were reported in France from 2000 to 2008 [14]. Japanese data (2007–2018) listing the bacteria responsible for TTBI reported no detection of *Pseudomonas* spp. [15]. Unfortunately, details regarding the bacterial load, age of PC, and immune status of recipients were not available for these cases. Thus, the causality in relation to patient outcome and a distinct evaluation of consequences for the clinical relevance of *Pseudomonas* spp. remain indeterminate. *P. fluorescens*, which is known to have a lack of or prolonged detection at temperatures above 34 °C [2,17], has only been reported once [14]. This might suggest either underreporting due to the conditions of the microbiological control (35 °C) or no or limited growth of *Pseudomonas* spp. in PCs due to storage conditions.

The presence of microbial contaminants in pharmaceutical products primarily depends on the growth capability in a certain matrix, whereas the detection of contaminated products is influenced by the matrix of the culture media, microorganisms, AE and AN conditions, and the incubation temperature [21]. So far, previous studies demonstrating that an incubation temperature of 35 °C is too high for the growth of *P. fluorescens* and *S. paucimobilis* have not taken the capability of these bacteria to grow in a PC matrix into consideration. Other studies dealing with the bacterial culture TTD in PCs included *P. aeruginosa* but not *P. fluorescens* [22]. Here, we systematically analysed the growth kinetics of three species—*P. aeruginosa*, *P. fluorescence*, and *P. putida*—in PCs as a matrix using various different isolates and a dual-temperature approach.

The spiking experiments conducted for this study clearly showed that some strains of *Pseudomonas* spp. (*P. aeruginosa*: 4/5 isolates, *P. fluorescens*: 1/5 isolates, *P. putida*: 2/3 isolates) are capable of growing in PCs despite showing donor-dependent heterogeneous growth kinetics. However, with the exception of one strain, all remaining *Pseudomonas* strains, as well as *S. paucimobilis*, showed a faster TTD at 35 °C compared to 25 °C. Only the *P. fluorescens* ATCC 13252 strain, the predecessor of which is known to have a lack of or prolonged detection at temperatures above 34 °C [2,17], showed some unique growth kinetics compared to the other *Pseudomonas* strains. In contrast to prior studies, this strain was detected at both cultivation temperatures, and a considerably prolonged detection time of 150 h was only determined in one of the four different PC samples. Nonetheless, it was the only strain that demonstrated reverse growth kinetics with a faster TTD at 25 than 35 °C. The detection of *P. aeruginosa* and *P. putida*, if capable of growing in PCs at all, did not benefit from a reduced incubation temperature; in fact, the opposite was true. Similarly, a study by Mastronardi et al., which developed a proficiency testing programme for cultural screening methods, showed acceptable growth kinetics for *P. aeruginosa* using the standard temperature of the BacT/Alert automated culture system [23].

The absence of bacterial growth combined with a negative cultural detection could generally be explained by the auto-sterilization capability of donor-specific factors. The detection of species by culture lacking a detectable growth assumed a lack of bacterial proliferation capability under PC storage conditions. The failure of adequate inoculation is also a possibility since the low inoculation titres required contain a high risk of sampling error. However, this could almost be excluded for samples in which bacterial proliferation or cultural detection was observed in at least one donor sample because the same master suspension was used for all inoculations.

When considering the proliferation capabilities of *Pseudomonas* spp. in PCs from different donors, one can assume that these species are more affected by the influence of donor-, product-, species- and strain-specific factors on bacterial proliferation.

This aspect agreed with the general complexity of specifying mandatory needs for the detection of bacterial contamination to enhance platelet safety. Beyond that, a wide variety of microorganisms came under consideration for the contamination of PCs, and, emanating from an initial bacterial contamination in the order of 10–100 CFU immediately after donation, bacterial growth could help to categorise species- and/or strain-dependency into four different kinetics [24,25]: (a) bacteria with no capacity for proliferating in blood components and are eliminated via auto-sterilization; (b) bacteria that survive in blood components but have no capability of proliferating (persistence); (c) bacteria surviving in blood components showing an extended lag-phase followed by slow proliferation; and (d) bacteria immediately proliferating after a short lag phase. Due to all of these aspects, a long-lasting discussion regarding the possibilities of further enhancing the bacterial safety of PCs is still ongoing. The subjects of this discussion include the shelf life of PCs; the optimal sampling strategy, sampling volumes, culture conditions to be used; and the availability and applicability of rapid detection methods [4,26,27]. Despite all efforts often resulting in risk reduction, the bacterial contamination of PCs and a risk of serious transfusion reactions remains; so far, zero risk has proved unattainable using the currently available screening strategies and technologies [26]. Among other tools to enhance the microbiological safety of PCs, the introduction of a second incubation temperature has been discussed in the literature. The logistical efforts were of minor importance when dual-temperature screening was performed at the end of shelf life in parallel to standard microbiological sterility testing. The inoculation of another one or two culture bottles is can be performed easily and rapidly. Cost analyses are needed to consider the additional culture media and the instrumental costs for the second incubation temperature. This approach would further elucidate the occurrence of bacteria preferring lower incubation temperatures and is currently the subject of an ongoing study. This evaluation changes if dual-temperature screening should also be implemented as part of microbiological screening during the shelf life of PCs to achieve the greatest benefit in terms of patient safety. Logistical efforts and costs will increase since a significantly higher number of PCs need to be tested. Secondly, the volume of sample available for testing is limited since product specifications must be adhered to if the product will subsequently be transfused to a patient. Moreover, the question of the optimal sampling strategy also affects this approach. Bacterial contamination resulting in TTBI is a rare event, and the overall incidence of bacterially contaminated PCs is relatively low.

So, is it worth the effort to establish a second incubation temperature for the microbiological control of PCs? Is there an unacceptable risk regarding potential adverse outcomes due to false-negative results or extended incubation periods, which could have repercussions on patient safety and regulatory compliance? Based on our current knowledge and the fact that, in this study, only one single isolate showed reduced cultural detection (not even failure) at a standard incubation temperature, we presently conclude that the costs and efforts required for the dual-temperature screening approach presented herein were not justified.

## Figures and Tables

**Table 1:** Overview of isolates *Enterobacteriacae*, *Bacillus* spp., *Staphylococcus* spp., *Streptococcus* spp.

Strain	Origin	Inoculum (CFU/PC)	Proliferation (*n*)	Detection Time				Figure
				25 °C (h (±SD))		35 °C (h (±SD))		
				AE	AN	AE	AN	
*Enterobacteriacae*								
*K. pneumoniae* ATCC 700603	RS	11–14	yes (3/3)	3.94 (±0.18)	4.01 (±0.22)	3.77 (±0.08)	3.60 (±0.55)	Figure 1A
*K. pneumoniae* ATCC 12633	RS							
*K. pneumoniae* PEI-B-0808 **	RS							
*E. coli* ATCC 25922	RS	6–12	yes (3/3)	4.24 (±0.19)	4.14 (±0.10)	3.77 (±0.07)	3.76 (±0.08)	Figure 1A
*E. coli* ATCC 35218	RS							
*Bacillus* spp.								
*B. cereus* ms12449	PC	10–30	yes (3/3)	5.46 (±0.90)	5.51 (±1.01)	4.09 (±0.26)	4.00 (±0.17)	Figure 1A
*B. cereus* ms9310	PC							
*B. cereus* ms13074	PC							
*B. subtilis* ATCC 6633 *	RS	10	yes (3/3)	18.22 (±4.06)	50.50 (±24.82)	9.65 (±1.69)	29.99 (±10.46)	Figure 1A
*Staphylococcus* spp.								
*S. epidermidis* 2427	PC	10–20	yes (3/3)	28.69 (±5.76)	29.00 (±7.81)	12.19 (±1.49)	12.14 (±1.89)	Figure 1C
*S. epidermidis* ms5106	PC							
*S. epidermidis* spr14021	PC							
*S. epidermidis* ms6031	PC							
*S. epidermidis* spr3907	PC							
*S. epidermidis* 186	PC							
*S. hominis* 2199	PC	13–18	yes (3/3)	26.79 (±4.37)	49.10 (±26.46)	10.41 (±1.22)	14.85 (±4.65)	Figure 1C
*S. hominis* 4224	PC							
*S. aureus* ms8401	PC	10	yes (3/3)	9.93 (±2.84)	9.70 (±3.25)	5.78 (±1.53)	5.93 (±1.73)	Figure 1B
*S. aureus* ATCC 6538	RS							
*S. aureus* ATCC 6581	RS							
*Streptococcus* spp.								
*S. mitis/S. oralis* spr 5920	PC	22–56	yes (3/3)	41.35 (±13.29)	37.35 (±8.54	17.38 (±4.58)	14.49 (±2.30)	Figure 1A
*S. mitis* 241	PC			30.56 (±4.47)	27.06 (±3.73)	12.79 (±1.23)	10.63 (±1.08)	
*S. mitis* 280	PC			48.51 (±4.61)	20.30 (±2.51)	9.33 (±0.84)	8.21 (±0.68)	
*S. pyogenes* ATCC 19615 *	RS	10	yes (3/3)	6.89 (±0.26)	6.67 (±0.17)	4.45 (±4.45)	4.23 (±4.23)	Figure 1A
*S. agalactiae* 8514	PC	16	yes (3/3)	6.85 (±2.43)	6.64 (±2.26)	4.69 (±0.85)	4.58 (±0.95)	Figure 1A

RS: reference stock, PC: PC isolate. * strain requested by the Eur. Ph. [1]. ** *Klebsiella pneumoniae* PEI-B-08-08 (conform to PEI-B-P-08, Transfusion-Relevant Bacteria References Strains TRBRS [16]).

**Table 2:** Overview of isolates *Pseudomonas* spp. and others.

Strain	Origin	Inoculum (CFU/PC)	Proliferation (*n*)	Detection Time				Figure
				25 °C (h (±SD))		35 °C (h (±SD))		
				AE	AN	AE	AN	
*Pseudomonas* spp.								
*P. aeruginosa* ATCC 9027	RS	12	no (5/5)	negative (5/5)				none
*P. aeruginosa* DSM 1128	RS	24	no (5/5)	24.23	43.25	14.25	43.05	Figure 2A
*P. aeruginosa* ATCC 27853	RS	24	yes (4/5)	16.11 (±4.81)	30.02 (±22.40)	10.21 (±2.42)	23.40 (±6.13)	Figure 2A
*P. aeruginosa* NTC 10662	RS	10	yes (5/5)	15.38 (±6.71)	35.72 (±14.39)	9.48 (±2.85)	20.32 (±4.80)	Figure 2A
*P. aeruginosa* L0120708	RS	21	yes (5/5)	13.60 (±4.88)	49.75 (±8.11)	8.81 (±2.49)	26.31 (±7.12)	Figure 2A
*P. fluorescens* DSM 6147	RS	54	no (3/3)		negative (3/3)			none
*P. fluorescens* DSM 50091	RS	12	no (3/3)		negative (3/3)			none
*P. fluorescens* DSM 50415	RS	67	no (3/3)		negative (3/3)			none
*P. fluorescens* ATCC 13525	RS	30–35	yes (4/5)	10.92 (±3.62)	negative	42.74 (±71.02)	negative	Figure 2B
*P. putida* DSM 6521	RS	24	yes (2/5)	7.81 (±0.93)	negative	6.20 (±1.25)	(20.27, 1 bottle)	Figure 2B
*P. putida* RV	P	24	yes (1/3)					Figure 2B
*P. putida* DSM 50257	RS	25	no (3/3)	negative (3/3)				none
*P. putida* ATCC 12633	RS	30–42	no (3/3)	negative (3/3)				none
Others								
*C. acnes* spr4694	PC	10–48	no (3/3)	statistical analysis not reliable due to heterogenous results				none
*C. acnes* spr14472	PC							
*C. acnes* spr4991	PC							
*C. acnes* ATCC 11827	RS							
*S. paucimobilis* DSM 1098	RS	10	yes (3/3)	44.41 (±13.0)	(28.35, 1 bottle)	36.25 (±8.15)	negative	Figure 1A
*C. minitissimum* ms10973	PC	30	no (3/3)	122.8 (±2.08)	negative	28.64 (±1.86)	negative	Figure 1C
*M. lutens is001*	P	16	no (3/3)	statistical analysis not reliable due to heterogenous results				Figure 1C

RS: reference stock, PC: PC isolate, P: isolate from hospitalized patient.

## Data Availability

The data of all results in this study are included in the manuscript. Raw data will be provided by the corresponding author upon reasonable request.
